# Corneal Wound Healing Requires IKB kinase β Signaling in Keratocytes

**DOI:** 10.1371/journal.pone.0151869

**Published:** 2016-03-17

**Authors:** Liang Chen, Maureen Mongan, Qinghang Meng, Qin Wang, Winston Kao, Ying Xia

**Affiliations:** 1 Department of Environmental Health and Center of Environmental Genetics, University of Cincinnati Medical Center, Cincinnati, Ohio, 45267–0056, United States of America; 2 Department of Ophthalmology, University of Cincinnati Medical Center, Cincinnati, Ohio, 45267–0056, United States of America; University of Oklahoma Health Sciences Center, UNITED STATES

## Abstract

IkB kinase β (IKKβ) is a key signaling kinase for inflammatory responses, but it also plays diverse cell type-specific roles that are not yet fully understood. Here we investigated the role of IKKβ in the cornea using *Ikkβ*^*ΔCS*^ mice in which the *Ikkβ* gene was specifically deleted in the corneal stromal keratocytes. The *Ikkβ*^*ΔCS*^ corneas had normal morphology, transparency and thickness; however, they did not heal well from mild alkali burn injury. In contrast to the *Ikkβ*^*F/F*^ corneas that restored transparency in 2 weeks after injury, over 50% of the *Ikkβ*^*ΔCS*^ corneas failed to fully recover. They instead developed recurrent haze with increased stromal thickness, severe inflammation and apoptosis. This pathogenesis correlated with sustained myofibroblast transformation with increased α smooth muscle actin (α-SMA) expression, higher levels of senescence β-Gal activity and scar tissue formation at the late stage of wound healing. In addition, the *Ikkβ*^*ΔCS*^ corneas displayed elevated expression of hemo-oxygenase-1 (HO-1), a marker of oxidative stress, and activation of stress signaling pathways with increased JNK, c-Jun and SMAD2/3 phosphorylation. These data suggest that IKKβ in keratocytes is required to repress oxidative stress and attenuate fibrogenesis and senescence in corneal wound healing.

## Introduction

IκB kinase β (IKKβ) is a key catalytic subunit of the IKK complex [1]. It plays a crucial role in the activation of NF-κB, which is a transcription factor that binds to κB elements in promoters and enhancers of target genes [2]. Stress stimuli can activate the IKKβ-NF-κB cascade, leading to either activation or repression of gene expression in a highly cell type-specific fashion. In immune cells, i.e. neutrophils and macrophages, this cascade leads to induction of genes coding for cytokines, chemokines, enzymes and molecules with microbicidal activity [3]. The immune cell IKKβ, therefore, plays a crucial role in protection against dangerous environmental stimuli. Although IKKβ is ubiquitously expressed in essentially all mammalian organisms, its role in non-immune cells is less well understood.

With the advent of genetic mutant mice in which the *Ikkβ* gene is deleted in specific cell types, it has become evident that IKKβ has diverse roles in the regulation of homeostasis, stress responses, survival and apoptosis [4]. Studies in mutant mice have shown that the IKKβ is required to maintain homeostasis of the immune responses in skin [5,6], to inhibit sensory excitability in neurons [7], to repress proliferation in hepatocytes [8], and to potentiate apoptosis in mammary epithelial cells, leading to mammary gland involution [9]. The physiological effects of IKKβ could be the consequence of modulation of tissue homeostasis and cell-cell interactions. The intestinal epithelial IKKβ, for example, protects the intestinal tract from bacterial infection via the suppression of local inflammation and improvement of epithelial cell survival [10]. Similarly, the hepatocyte IKKβ prevents chemical carcinogenicity by reducing hepatocyte ROS accumulation and apoptosis and alleviating the activation of liver macrophages [11].

The cornea consists of five distinct layers: a stratified non-keratinized epithelial cell layer, the Bowman’s membrane, a highly organized collagenous stroma layer interspersed with keratocytes, the Descemet’s membrane and a single endothelial cell layer [12]. Previously, we used gene knockout approach and investigated the role of IKKβ in corneal epithelial cells [13]. We showed that IKKβ is dispensable for pre- and post-natal corneal epithelium development, but is required for optimal healing of corneal epithelial debridement wounds. Mechanistically, IKKβ is required for activation of the NF-κB and p38 signaling pathways, which lead to corneal epithelial cell migration for wound healing.

Here we have applied the similar approach to characterize the roles of IKKβ in keratocytes, the residential cells of the corneal stroma. We show that the keratocyte IKKβ is also dispensable for corneal development, but is required for wound healing. In response to mild alkaline burn injury [14], IKKβ-deficient corneas exhibit defective healing associated with excess ROS, stress signaling pathway activation, myofibroblast transformation and senescence. These results suggest that the keratocyte IKKβ modulates multiple stress signaling pathways in corneal wound healing responses.

## Materials and Methods

### Mouse strains, reagents and antibodies

The *Ikkβ*^*F/F*^ mice were a gift from Dr. Michael Karin at the University of California at San Diego and the *Kera-Cre* mice were described before [15], The mice (n = 94) used in this work were housed in the Experimental Animal Laboratory at the University of Cincinnati. The procedures carried out in this work are in strict accordance with the recommendations in the Guide for the Care and Use of Laboratory Animals of the National Institutes of Health. The protocol (no. 06-04-19-01) approved by the Institutional Animal Care and Use Committee (IACUC) of the University of Cincinnati. Euthanasia was done by carbon dioxide (CO2) to effect followed by cervical dislocation.

The following antibodies were used in the study: anti-p-SMAD 2 (Ser-465, 467), anti-SMAD 3 (Ser-423, 425) and anti-p-Jun (Ser 63, 73) were from Cell Signaling, anti-α-SMA from Abcam, anti-β-actin from Sigma-Aldrich, anti-CD45 and anti-CD11b from Invitrogen, anti-p-JNK (Thr-183, Tyr-185) from Promega, and anti-HO-1 from StressGen Biotechnologies

### *In vivo* alkali burn of the cornea

Alkali burn corneal injuries were done following protocols described before with minor modifications [14]. Briefly, animals were anesthetized by intraperitoneal injection of ketamine hydrochloride (80 mg/kg) and xylazine (10 mg/kg). Ocular surface alkali burns were produced by placing 3MM chromatography paper (Whatman) cut into 2-mm diameter circles previously soaked in 0.05 N NaOH onto the central cornea for exactly 90 seconds. The eyes were continuously washed with PBS for 1 min, terramycin ophthalmic ointment was topically administered to the eyes, and the animals were placed on a warming pad. At least 8 mice were used under each experimental condition.

### Evaluation of corneal opacity, histological and immunohistochemical analysis

Corneal opacity was evaluated by stereoscopic microscopy and slit lamp. The opacity was scored as: 0, completely clear cornea, +1, slight opacity, +2, mild opacity with iris and lens visible, +3, severe opacity with iris and lens invisible, but opacity was limited to the cauterized area, +4, extensive opacity throughout the entire cornea. Cryosections of the eye tissues were subjected to H&E staining following standard procedures. Immunohistochemical analysis was done as described previously [13]. TUNEL was done using the ApopTag Plus In Situ Apoptosis Fluorescein Detection Kit in accordance to the manufacture’s instruction (Millipore). The SA-β-Gal activities were measured at the pH 6.0 using Beta-Glo Assay system (Promega). Stained sections were mounted and photographed using an Axio Observer Inverted Microscope (Carl Zeiss).

### Statistical analyses

The data were analyzed by either two-tailed student *t*-test or ANOVA. * *p*< 0.05, ** *p*< 0.01 and *** *p*< 0.001 were considered statistically significant.

## Results

### Generation of *Ikkβ* keratocyte knockout mice

**Fig 1:**
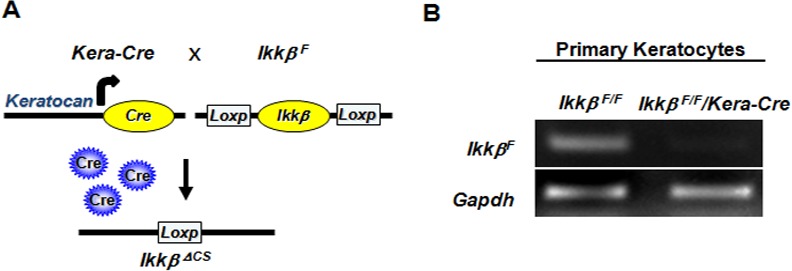
Generation of *Ikkβ*^*ΔCS*^ mice. (A) Schematic illustration of the strategy for generating corneal stroma-specific *Ikkβ* knockout mice, namely *Ikkβ*^*ΔCS*^ mice. The *Kera-Cre* transgenic mice, in which the Cre recombinase gene was controlled by the *Keratocan* promoter, were crossed with *Ikkβ*^*F/F*^ mice. In the *Kera-Cre/Ikkβ*^*F*^, *i*.*e*. *Ikkβ*^*ΔCS*^ mice, the *Ikkβ* floxed alleles were ablated specifically in the corneal stromal keatocytes. (B) Genomic DNA of the corneal stroma isolated from the *Ikkβ*^*F*^ and *Ikkβ*^*ΔCS*^ mice was genotyped by PCR using primers specific for the *Ikkβ*^*F*^ allele and *Gapdh*.

The *Kera-Cre* transgenic mice carry Cre recombinase gene controlled by the *Keratocan* promoter [16]. We crossed *Ikkβ*^*F/F*^ and *Kera-Cre* mice and identified the *Ikkβ*^F/F^ and Kera-Cre genes in the offspring by genotyping of tail genomic DNA (Fig 1A). To evaluate the efficiency of *Ikkβ* ablation, we isolated corneal stromal cells from adult eyes, extracted genomic DNA, and performed PCR using primers amplifying the *Ikkβ*^F^ allele. While the products of PCR amplification were detected in cells isolated from *Ikkβ*^*F/F*^ corneas, they were absent in cells isolated from *Ikkβ*^*F/F*^/Kera-Cre corneas, though *Gapdh* used as control was amplified in both cells (Fig 1B). These data confirmed that IKKβ was successfully ablated in the corneal stroma of *Ikkβ*^F/F^/Kera-Cre mice, henceforth referred to as *Ikkβ*^*ΔCS*^.

### Normal corneal development of *Ikkβ*^*ΔCS*^ mice

**Fig 2:**
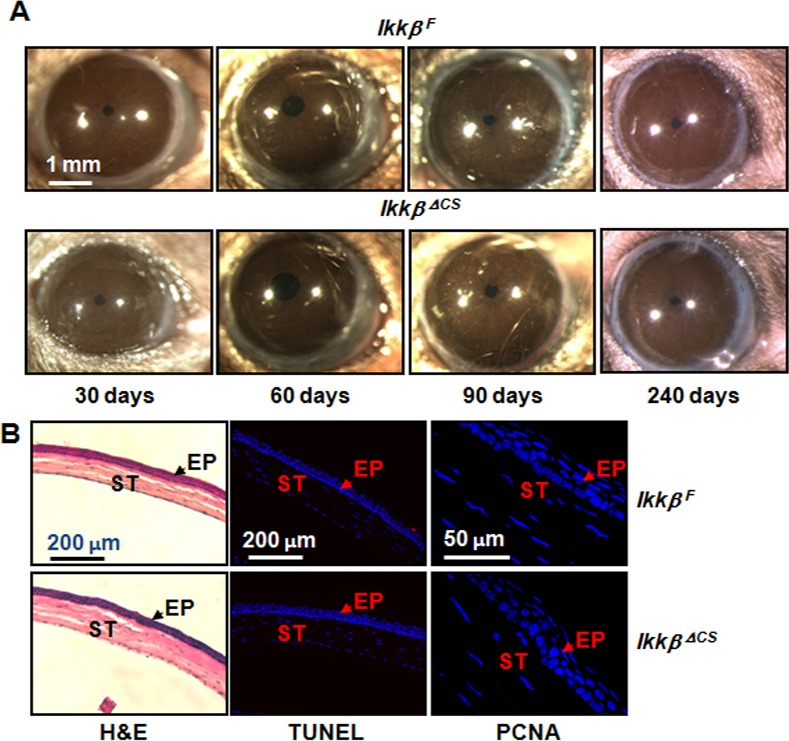
Role of IKKβ in corneal development and maintenance. (A) The eyes of *Ikkβ*^*F*^ and *Ikkβ*^*ΔCS*^ mice were examined under a stereoscope and photographed. (B) The *Ikkβ*^*F*^ and *Ikkβ*^*ΔCS*^ eyes were analyzed by H&E staining, TUNEL assay and immunostaining using anti-PCNA. Blue: Hoechst for nuclei. Red: TUNEL and PCNA positive signals, which were absent in the cornea of adult mice. ST: corneal stroma, EP, corneal epithelium, labeled with arrows. Pictures represent results from at least 3 mouse corneas of each genotype.

To assess if the keratocyte IKKβ might be required for corneal development, we used stereoscopic examination of the eyes of *Ikkβ*^*F*^ and *Ikkβ*^*ΔCS*^ mice from 1 month- up to 8 months-old. The gross morphology and transparency of the eyes of *Ikkβ*^*ΔCS*^ mice were indistinguishable from those of *Ikkβ*^*F*^ mice (Fig 2A). *Ikkβ*^*F*^ and *Ikkβ*^*ΔCS*^ adult eyes had also similar corneal thickness as determined by histological examination after H&E staining (Fig 2B). Neither the *Ikkβ*^*F*^ nor the *Ikkβ*^*ΔCS*^ corneas had obvious evidence of cell proliferation or apoptosis as shown by PCNA staining and Terminal deoxynucleotidyl transferase dUTP nick end labeling (TUNEL), respectively. Hence, IKKβ expression in keratocytes is dispensable for corneal development and maintenance.

### IKKβ is required for optimal corneal wound healing

**Fig 3:**
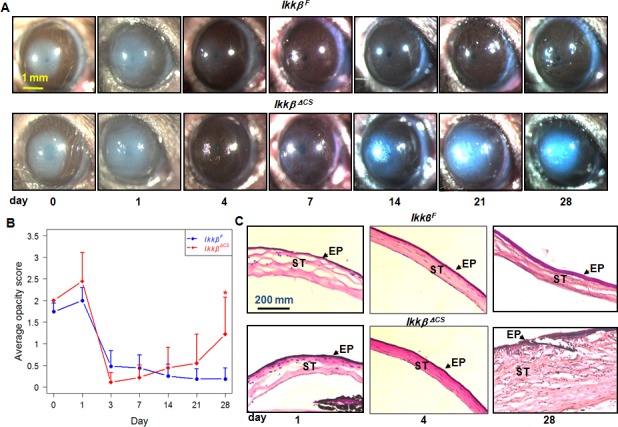
Corneal wound healing in *Ikkβ*^*F*^ and *Ikkβ* ^*ΔCS*^ mice. The *Ikkβ*^*F*^ and *Ikkβ* ^*ΔCS*^ mice were subjected to mild alkali burn corneal injury, and the eyes were examined under a stereoscope and slit lamp. (A) Representative photos of the eyes at different days after injury, and (B) the average opacity score are presented as mean±SEM of at least 8 eyes examined under each genotype/experimental conditions. Significant differences between groups were calculated using 2-way repeated measures ANOVA followed by post hoc multiple comparisons of means (Tukey method), and *p < 0.05 is considered statistically significant between the genotypes on the given days of injury. (C) The wounded eyes were harvested at different days after injury and examined by H&E. ST: corneal stroma, EP, corneal epithelium, labeled with arrows.

To evaluate whether keratocyte IKKβ was required for wound healing after environmental insults [17], we performed mild alkali burn injury on the *Ikkβ*^*F*^ and *Ikkβ*^*ΔCS*^ corneas and examined healing stereoscopically. Both *Ikkβ*^*F*^ and *Ikkβ*^*ΔCS*^ eyes displayed haze at 1 day post-injury and gradual resolution of the haze 4–7 days after injury (Fig 3A). However, while all of the *Ikkβ*^*F*^ corneas restored transparency at 2 weeks after injury, approximately half of the *Ikkβ*^*ΔCS*^ corneas (4 out of 8) had recurrent haze and became cloudy. The opacity score was high at day 0–1 and reduced at 4–14 days after injury in all of the eyes examined. It remained low in *Ikkβ*^*F*^, but became significantly higher in *Ikkβ*^*ΔCS*^ corneas at 4 weeks after injury (Fig 3B). Histological examination showed that the *Ikkβ*^*F*^ and *Ikkβ*^*ΔCS*^ corneas were not much different at 1 and 4 days after injury, but at 28 days, though the *Ikkβ*^*F*^ corneas showed normal morphology (data not shown), the cloudy *Ikkβ*^*ΔCS*^ corneas were swollen with increased stroma thickness (Fig 3C). These *Ikkβ*^*ΔCS*^ corneas also exhibited severe epithelium disruption, epithelial cell protrusion into stroma and scar tissue formation.

### Inflammatory and stress responses in *Ikkβ*^*ΔCS*^ corneas

**Fig 4:**
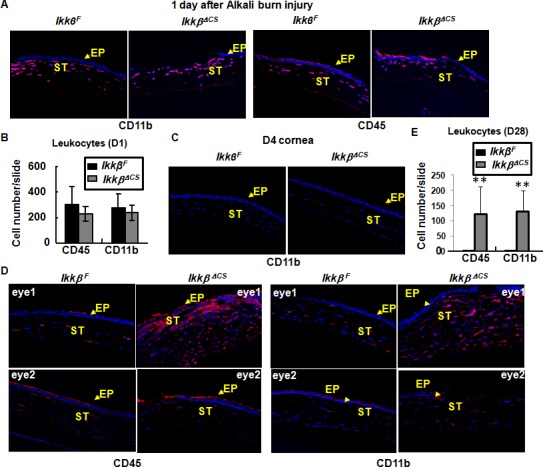
Inflammation in the injured cornea. The *Ikkβ*^*F*^ and *Ikkβ* ^*ΔCS*^ eyes were harvested at (A and B) 1 day, (C) 4 days, and (D and E) 28 days after alkali burn injury. The tissues were processed and used for immunohistochemistry using anti-CD11b and anti-CD45 for infiltrated leukocytes. Blue: DAPI (nuclei), Red: leukocytes. (D) Of the two *Ikkβ* ^*ΔCS*^ eyes examined, only eye1 was opaque. The number of leukocytes in the eyes at (B) 1 day and (E) 28 days after injury was quantified. Data represent average values from at least 5 slides/eye and 3 injured eyes examined. ST: corneal stroma, EP, corneal epithelium, labeled with arrows. **p<0.01 was considered significantly different between *Ikkβ*^*F*^ and *Ikkβ* ^*ΔCS*^ eyes.

Alkali burn injury of the cornea evokes inflammatory responses and cellular stress. Inflammatory responses facilitate tissue remodeling essential for wound healing, but if excessive, they obstruct healing and cause severe damage [18]. Given IKKβ’s role in inflammatory signaling, we hypothesized that its ablation in keratocytes would perturb corneal inflammation. The injured corneas were examined by immunohistochemistry using anti-CD11b that detects macrophages and anti-CD45 that detects neutrophils. At the early phase of wound healing, the *Ikkβ*^*F*^ and *Ikkβ*^*ΔCS*^ corneas were similar, with massive macrophages and neutrophils at 1 day, but no inflammatory cells detected at 4 days after injury (Fig 4A–4C). At the late phase, i.e. 28 days, the *Ikkβ*^*F*^ corneas (eye 1 and eye 2) had a few detectable inflammatory cells; however, the opaque *Ikkβ*^*ΔCS*^ corneas (eye 1) were filled with macrophages and neutrophils (Fig 4D). On the other hand, if the *Ikkβ*^*ΔCS*^ corneas were transparent (eye 2) at 28 days, they were similar to the *Ikkβ*^*F*^ eyes with little, if any, detectable inflammatory cells. The number of inflammatory cells in *Ikkβ*^*ΔCS*^ corneas was more abundant than that in *Ikkβ*^*F*^ corneas (Fig 4E). These observations suggest that IKKβ may prevent corneal opacity by facilitating resolution of inflammation at the late phase of wound healing.

**Fig 5:**
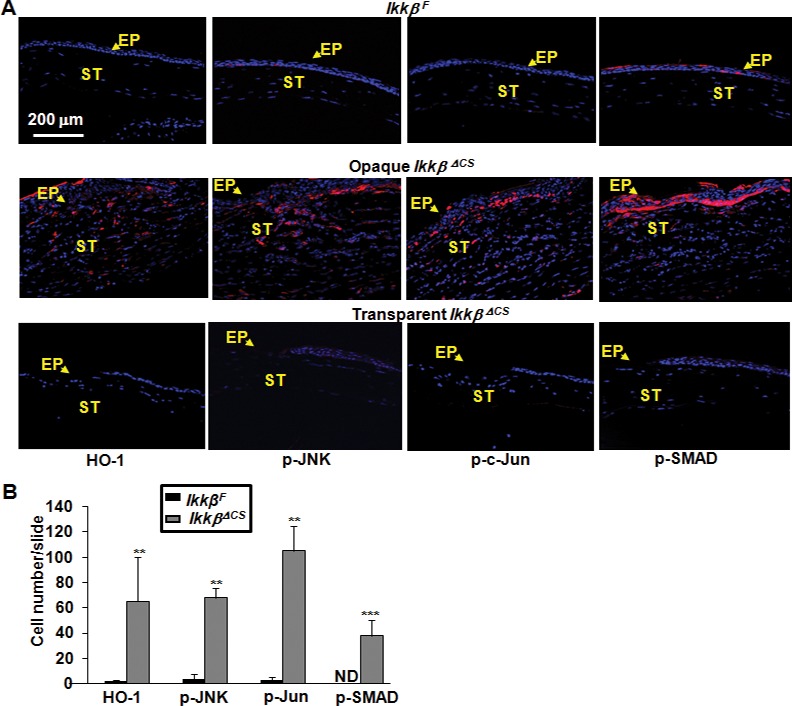
Oxidative stress and stress signaling in the injured cornea. The *Ikkβ*^*F*^ and *Ikkβ* ^*ΔCS*^ eyes were harvested at 28 days after alkali burn injury. The tissues were processed and used for immunohistochemistry using anti-HO-1, a marker of oxidative stress, anti-pJNK and p-c-Jun, markers for the stress-activated JNK pathway, and anti-p-SMAD, a marker for active TGFβ signaling. Blue: DAPI (nuclei), Red: leukocytes. ST: corneal stroma, EP, corneal epithelium, labeled with arrows. (B) The number of positive cells was quantified and **p<0.01 and ***p<0.001 was considered significantly different between *Ikkβ*^*F*^ and *Ikkβ* ^*ΔCS*^ eyes. Results represent at least 5 slides/eye and 3 injured eyes examined.

Besides its role in inflammatory signaling, the IKKβ is known to be involved in the modulation of redox homeostasis; its ablation has been linked to severe oxidative stress and tissue injury, which may in turn potentiate inflammation and cytotoxicity [11,19,20]. We therefore examined whether IKKβ ablation affected the stress responses by measuring the expression of hemeoxygenase-1 (HO-1), encoded by an oxidative stress-inducible gene. In contrast to the *Ikkβ*^*F*^ corneas, which did not have any detectable HO-1, the opaque, but not transparent, *Ikkβ*^*ΔCS*^ corneas had abundant HO-1 expression at 28 days of injury (Fig 5A and 5B). It is known that elevated oxidative stress can lead to the activation of stress-induced signaling pathways, such as the JNK-c-Jun cascade [21]. Indeed, the expression of HO-1 is accompanied by the activation of stress markers, e. g., p-JNK, p-C-Jun (Fig 5). In addition, the opaque *Ikkβ*^*ΔCS*^ corneas had increased phosphorylation of SMAD, markers of TGFβ signaling [22–24]. The *Ikkβ*^*F*^ and transparent *Ikkβ*^*ΔCS*^ corneas, in contrast, did not have any detectable p-SMAD. Taken together, the aberrant wound healing responses in the *Ikkβ*^*ΔCS*^ corneas correspond to sustained inflammation with concurrent increase of oxidative stress and activation of the stress signaling pathways.

### Cellular activities affected by IKKβ ablation

**Fig 6:**
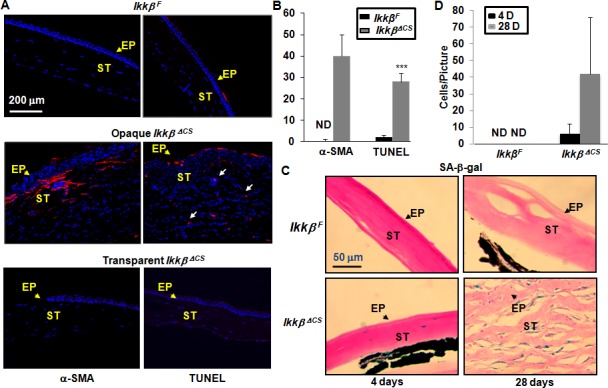
Myofibroblast transformation, senescence and apoptosis of the injured cornea. (A) The *Ikkβ*^*F*^ and *Ikkβ* ^*ΔCS*^ eyes were harvested at 28 days after alkali burn injury. The tissues were processed and used for immunohistochemistry using anti-α-SMA, a marker for myofibroblast and TUNEL assays for the detection of apoptotic cells. Blue: DAPI (nuclei), Red: leukocytes. (B) The *Ikkβ*^*F*^ and *Ikkβ* ^*ΔCS*^ eyes were harvested at 4 days and 28 days after alkali burn injury. The tissue sections were examined by SA-β-Gal staining. The SA-β-Gal positive cells are stained with blue color. ST: corneal stroma, EP, corneal epithelium, labeled with arrows. (B and D) The number of staining positive cells was quantified and **p<0.01 and ***p<0.001 was considered significantly different between *Ikkβ*^*F*^ and *Ikkβ* ^*ΔCS*^ eyes. Data represent at least at least 5 slides/eye and 3 injured eyes examined.

TGFβ promotes myofibroblast transformation in corneal wound healing [25,26]. The finding that TGFβ signaling was upregulated in the *Ikkβ*^*ΔCS*^ corneas prompted us to examine the expression of α smooth muscle actin (α-SMA), a marker of myofibroblasts. There was indeed abundant α-SMA expression in the opaque *Ikkβ*^*ΔCS*^, but not in the *Ikkβ*^*F*^ and transparent *Ikkβ*^*ΔCS*^ corneas at 28 days after injury (Fig 6A and 6B).

Both TGFβ activation and sustained oxidative stress can induce, stabilize and amplify senescence, leading to the detrimental effects of cell death [27,28]. The expression of senescence-associated β-Galactosidase (SA-β-Gal) [29], although completely undetectable in the *Ikkβ*^*F*^ corneas, was detected in a few cells in the *Ikkβ*^*ΔCS*^ corneas at 4 days after injury (Fig 6C). While the SA-β-Gal activity remained undetectable in the *Ikkβ*^*F*^ corneas at 28 days after injury, it was markedly amplified in the opaque *Ikkβ*^*ΔCS*^ corneas (Fig 6C and 6D). Concurrently, the opaque, but not transparent *Ikkβ*^*ΔCS*^ corneas displayed increased apoptosis, detected by TUNEL staining (Fig 6A and 6B). Our data suggest that IKKβ expression in keratocytes is required for the repression of fibrogenesis, senescence and apoptosis in corneal wound healing.

## Discussion

In the present work, we show that IKKβ expression in keratocyte is dispensable for corneal development, but required for optimal wound healing. We and others have previously shown that IKKβ expression in fibroblasts is essential to maintain redox homeostasis, and it does so through NF-κB, which regulates anti-oxidant gene expression [20,30–32]. Data presented here suggest that IKKβ has a similar role in keratocytes, the cornea-specific fibroblasts [17]. *Ikkβ*^*ΔCS*^ corneas exhibit elevated oxidative stress and activation of stress signaling pathways after stromal injury. In contrast to wild type corneas, which eventually recover from mild alkaline burn injury, many of the *Ikkβ*^*ΔCS*^ corneas become cloudy and swollen with scar formation. Our data are consistent with the notion that excessive oxidative stress impede the healing of corneal stromal wounds [33,34].

The corneal keratocytes are quiescent in the absence of external insults, but enter cell cycle and become active under pathologic conditions [35,36]. In response to injury, the keratocytes differentiate to myofibroblasts essential for contraction and wound closure; excessive myofibroblast transformation, on the other hand, will result in fibrosis and scars [37,38]. The *Ikkβ*^*ΔCS*^ corneas have sustained myofibroblast activation defined by the expression of α-SMA, and correspondingly, they exhibit strong activation of the TGFβ pathway, a potent inducer of myofibroblast differentiation [39]. Interestingly, *Ikkβ*^*-/-*^ fibroblasts display similar phenotype (Chen, et. al., data not shown). Studies in fibroblasts have shown that loss of IKKβ leads to oxidative stress, which induces c-Jun binding and activation of the *Tgfβ* promoter and gene expression; TGFβ in turn potentiates myofibroblast transformation and senescence (Chen, et. al., data not shown). It is possible that oxidative stress also serves as a molecular link between IKKβ and TGFβ signaling in the *Ikkβ*^*ΔCS*^ corneas.

Corneal wound healing involves an early inflammatory phase followed by a late remodeling phase. In the early phase, tissue damage triggers neutrophil infiltration and macrophage invasion. These inflammatory cells produce cytokines, chemokines and molecules with microbicidal activity important for protecting the cornea from infection and environmental insults. Previous studies by Saika, et. al. have shown that activation of the IKK-NF-κB pathways in the neutrophil and macrophage makes the major contribution to the inflammatory responses in corneal wound healing [40]. Consistent with this notion, we show that IKKβ in keratocytes is not required for early phase inflammatory responses, but instead seems to be involved in the maintenance of tissue homeostasis. In fact, IKKβ exhibits similar functions in other non-immune cells [41], such as hepatocytes [11], keratinocytes [5] and intestinal epithelial cells [42].

The mechanisms through which IKKβ regulates redox homeostasis and TGFβ signaling have been investigated in fibroblasts. In essence, IKKβ is required for optimal expression of redox scavengers, and IKKβ-null cells have decreased capacity to counteract oxidative stress elicited by environmental insults. When oxidative stress increases to a threshold level, it activates the JNK-c-Jun pathway, which induces TGFβ expression and activity; TGFβ in turn can act through NADH oxidase to further potentiate oxidative stress. Due to activation of the autocrine amplification of the ROS-TGFβ-NOX loop, IKKβ ablation in fibroblasts leads to a progressive increase of oxidative stress and TGFβ signaling, and a gradual myofibroblast transformation and premature senescence (Chen, et. al. unpublished data). It is possible that IKKβ also regulates redox homeostasis in keratocytes, where the activation of the ROS-TGFβ-NOX loop leads to the more severe wound healing defects observed in the *Ikkβ*^*ΔCS*^ corneas.

## Supporting Information

S1 File Supplemental file for reviewers. (PDF)
